# Non - invasive right ventricular efficiency using 4D flow MRI

**DOI:** 10.1186/1532-429X-17-S1-Q58

**Published:** 2015-02-03

**Authors:** Alejandro Roldán-Alzate, Scott W Grogan, Heidi B Kellihan, Alessandro Bellofiore, Naomi C Chesler, Oliver Wieben, Christopher J Francois

**Affiliations:** 1 Radiology, University of Wisconsin, Madison, WI, USA; 2 Biomedical Engineering, University of Wisconsin, Madison, WI, USA; 3 Veterinary Medicine, University of Wisconsin, Madison, WI, USA

## Background

Pulmonary arterial hypertension (PH) is a progressive disease of increased resistance to flow through the lungs, leading to right ventricular (RV) failure [1]. MRI is increasingly used to assess right ventricular (RV) function in PH. RV stroke work (SW) based on invasive pressure and volume measurements, is used to assess ventricular work. Determining RV work from MRI could enable a more complete characterization of RV and PA interactions in PH. The purpose of this study was to non-invasively estimate RV work from simultaneously acquired RV volume (V_RV_) and pulmonary artery flow (Q_PA_) using a 4D flow-sensitive MRI sequence in a canine model.

## Methods

**Figure 1:**
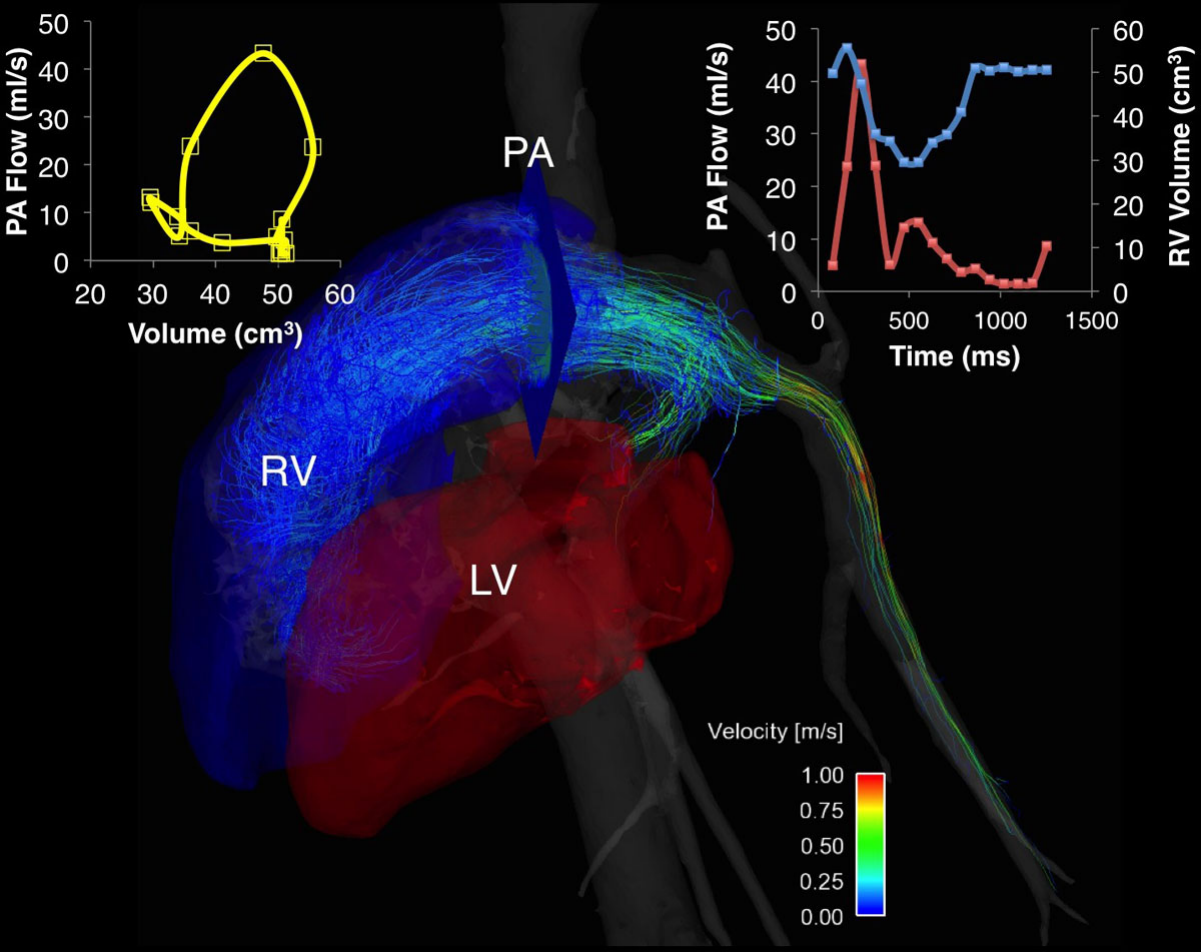
Visualization and quantification of pulmonary arterial flow and right ventricular volume using 4D Flow MR images.

After IACUC approval, hemodynamic measurements were performed prior to and following induction of acute PH by injection of embolizing micro-beads; details are available elsewhere [2]. Pre- and post-embolization right heart catheterization (RHC) was performed to measure hemodynamic changes in the RV and PA. 4D flow MRI (Phase Contrast with Vastly undersampled Isotropic Projection Reconstruction - PCVIPR) was performed on 3T clinical scanners (MR750, GE Healthcare, Waukesha, WI) after the intravenous administration of gadolinium-based contrast agents. PCVIPR parameters: FOV=32x32x22cm, isotropic 1.3mm spatial resolution, TR/TE=6.3/2.1ms, Venc=150cm/s, scan time: ~10min using adaptive respiratory gating of bellows and retrospective ECG gating [3]. Post-processing was done using Mimics (Materialise, Ann Arbor, MI) for the segmentation of the V_RV_ from dynamic magnitude images and Ensight (CEI, Apex, NC) for quantification of Q_PA_. RV pressure - V_RV_ loops were generated to assess SW by calculating the area inside the loop. Q_PA_ - V_RV_ loops were generated and their area calculated for comparison to the SW calculations (Fig 1). Direct comparison was used for the analysis of the results and a student ttest was used to compare the two methods.

## Results

**Figure 2:**
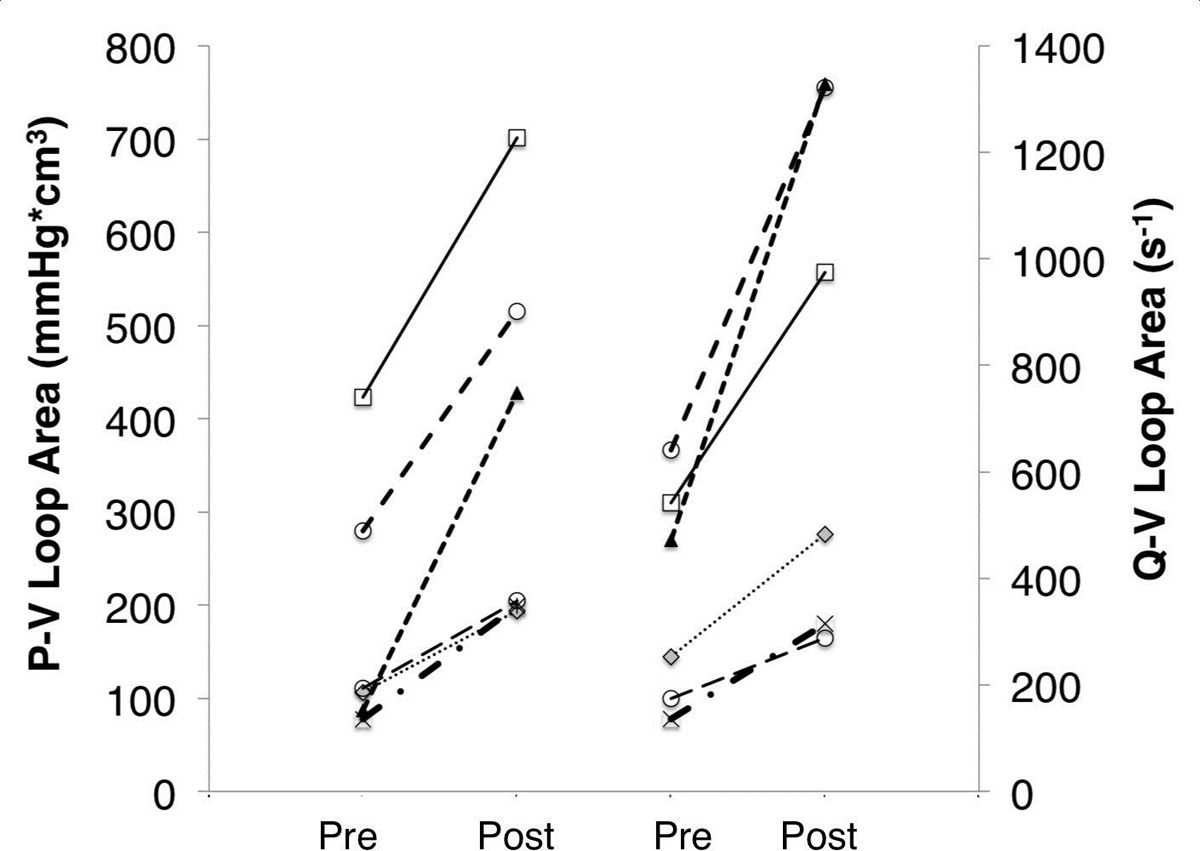
Direct comparison of P-V loop area and QMPA-V loop area before and after pulmonary embolization.

In all cases embolization induced an increase in SW (180 ± 140 vs 374 ± 210mmHg*cm^3^). Similarly the calculated area of the Q_PA_ - V_RV_ loops increased for all the cases (369 ± 210 vs 785 ± 486 s^-1^) (Fig 2). No significant difference was found between the percent increase of SW and the Q_PA_ - V_RV_ loops area (53 ± 15 vs 52 ± 12%, p = 0.95).

## Conclusions

Q_MPA_ - V_RV_ loop area estimated noninvasively using 4D flow MRI can be used to evaluate right ventricular stroke work. The results from this study indicate that 4D flow-sensitive MRI with PC VIPR can also be used to estimate right ventricular work, complementing the analysis of alterations in flow patterns in the heart and pulmonary arteries in patients with cardiopulmonary disease, however more studies need to be done for validation of the model.

## Funding

NIH R01HL072260, NIH R01HL086939, and the Department of Radiology.
